# Supplementary material for: Accuracy of Computer-Assisted Surgery in Maxillary Reconstruction: A Systematic Review
Source: J Clin Med. 2021 Mar 16;10(6):1226. doi: 10.3390/jcm10061226 (PMC8002284; doi:10.3390/jcm10061226)
Supplement: Supplementary file 1 [file jcm-10-01226-s001.pdf]

Table S1. Search strategy accuracy of computer-assisted surgery in maxillary reconstruction.

Search	PubMed Query	Items found
#5	#1 AND #2 AND #3 AND #4	920
#4	"Reproducibility of Results"[Mesh] OR reproducibil*[tiab] OR accurac*[tiab] OR accurate[tiab] OR precis*[tiab] OR reliabl*[tiab] OR reliabil*[tiab] OR valid*[tiab] OR correct*[tiab] OR repeat*[tiab]	3185145
#3	"Surgery, Computer-Assisted"[Mesh] OR "Image Processing, Computer-Assisted"[Mesh] OR "Multimodal Imaging"[Mesh] OR imag*[tiab] OR three-dimension*[tiab] OR 3-D[tiab] OR 3-dimension*[tiab] OR 3D[tiab] OR digitali*[tiab] OR computer*[tiab] OR pre-operative plan*[tiab] OR preoperative plan*[tiab] OR (virtual[tiab] AND surgical[tiab] AND plann*[tiab]) OR vsp[tiab] OR rapid prototyp*[tiab] OR stereolithograph*[tiab] OR intraoperative navigat*[tiab] OR CAD/CAM[tiab] OR CAS[tiab] OR cutting guide*[tiab] OR surgical template*[tiab] OR mirror function*[tiab]	1887919
#2	"Reconstructive Surgical Procedures"[Mesh:NoExp] OR reconstruct*[tiab]	325896
#1	"Maxilla"[Mesh] OR maxillectom*[tiab] OR maxilla*[tiab] OR maxillo*[tiab] OR midface[tiab] OR palatomaxill*[tiab] OR hard palate[tiab] OR palatal[tiab] OR upper jaw[tiab] OR hemimaxill*[tiab]	118594

Search strategy in PubMed from 8 January 2021 (read from bottom-up).

Search	Embase.com Query	Items found
#6	#5 NOT ('conference abstract'/it OR 'conference review'/it OR 'editorial'/it OR 'letter'/it OR 'note'/it OR 'short survey'/it)	1714
#5	#1 AND #2 AND #3 AND #4	2257
#4	'data accuracy'/exp OR 'measurement precision'/exp OR 'measurement repeatability'/exp OR 'repeatability'/exp OR 'accuracy'/exp OR 'reliability'/exp OR reproducibil*:ab,ti,kw OR accurac*:ab,ti,kw OR accurate:ab,ti,kw OR precis*:ab,ti,kw OR reliabl*:ab,ti,kw OR reliabil*:ab,ti,kw OR valid*:ab,ti,kw OR correct*:ab,ti,kw OR repeat*:ab,ti,kw	4096457
#3	'image processing'/exp OR 'multimodal imaging'/exp OR 'computer assisted surgery'/exp OR 'three dimensional imaging'/de OR 'image reconstruction'/exp OR imag*:ab,ti,kw OR ‘three-dimension*’:ab,ti,kw OR 3-dimension*:ab,ti,kw OR 3D:ab,ti,kw OR 3-D:ab,ti,kw OR digitali*:ab,ti,kw OR computer*:ab,ti,kw OR ‘pre-operative plan*’:ab,ti,kw OR ‘preoperative plan*’:ab,ti,kw OR (virtual NEAR/3 surgical NEAR/3 plann*):ab,ti,kw OR vsp OR ‘rapid prototyp*’:ab,ti,kw OR stereolithograph*:ab,ti,kw OR ‘intraoperative navigat*’:ab,ti,kw OR ‘CAD/CAM’:ab,ti,kw OR CAS:ab,ti,kw OR ‘cutting guide*’:ab,ti,kw OR ‘surgical template*’:ab,ti,kw OR ‘mirror function*’:ab,ti,kw	2440940
#2	'reconstructive surgery'/exp OR 'craniofacial surgery'/exp OR reconstruct*:ab,ti,kw	435124
#1	'maxilla'/exp OR 'hard palate'/exp OR 'maxilla resection'/exp OR maxillectom*:ab,ti,kw OR maxilla*:ab,ti,kw OR maxillo*:ab,ti,kw OR midface:ab,ti,kw OR palatomaxill*:ab,ti,kw OR ‘hard palate’:ab,ti,kw OR palatal:ab,ti,kw OR ‘upper jaw’:ab,ti,kw OR hemimaxill*:ab,ti,kw	136690

Search strategy in Embase.com from 8 January 2021 (read from bottom-up).

Search	Cochrane Library Query	Items found
#5	#1 AND #2 AND #3 AND #4	43

Search	Cochrane Library Query	Items found
#4	(reproducibil* OR accurac* OR accurate OR precis* OR reliabl* OR reliabil* OR valid* OR correct* OR repeat*):ti,ab,kw (word variatons have been searched)	203513
#3	(imag* OR three NEXT dimension* OR 3 NEXT dimension* OR 3D OR 3 NEXT D OR digitali* OR computer* OR pre NEXT operative NEXT plan* OR preoperative NEXT plan* OR virtual NEAR/3 surgical NEAR/3 plann* OR vsp OR rapid NEXT prototyp* OR stereolithograph* OR intraoperative NEXT navigat* OR CAD NEXT CAM OR CAS OR cutting NEXT guide* OR surgical NEXT template* OR mirror NEXT function*):ti,ab,kw (word variatons have been searched)	149823
#2	reconstruct*:ti,ab,kw (word variatons have been searched)	10285
#1	(maxilla* OR maxillectom* OR maxillo* OR midface OR palatomaxill* OR “hard palate” OR palatal OR “upper jaw” OR hemimaxill*):ti,ab,kw (word variatons have been searched)	8151

Search strategy in The Cochrane Library from 8 January 2021 (read from bottom-up).

Table S2. Study design and risk of bias assessment.

Author	Study design	Assessment of bias	1	2	3	4	5	6	7	8	9	10	11	12	13	14	15	16	17	18	19	20
Liu et al. (2009) [18]	RCS	10/20	1	0	0	0	1	0	0	1	1	1	0	1	1	0	1	0	0	1	1	0
Melville et al. (2019) [19]	RCS	11/20	1	0	0	0	1	0	1	1	1	0	0	1	1	0	1	0	0	1	1	1
Morita et al. (2017) [20]	RCS	10/20	1	0	0	0	1	0	1	1	1	0	0	1	1	0	0	0	0	1	1	1
Numajiri et al. (2018) [21]	RCS	10/20	1	0	0	1	0	0	0	1	1	1	0	1	1	0	0	0	0	1	1	1
Schepers et al. (2016) [8]	RCS	12/20	1	0	0	1	1	0	1	1	1	1	0	1	1	0	0	0	1	0	1	1
Tarsitano et al. (2016) [22]	PCS	15/20	1	1	0	1	1	1	1	1	1	1	0	1	1	0	1	0	0	1	1	1
Yang et al. (2018) [23]	PCS	15/20	1	1	0	1	1	0	1	1	1	1	0	1	1	0	1	0	1	1	1	1
Zheng et al. (2016) [25]	RCS	11/20	1	0	0	1	1	0	0	1	1	1	0	1	1	0	1	0	0	1	1	0
Navarro Cuéllar et al. (2021) [27]	RCCS	18/24	2	2	0	2	0	2	2	0	2	2	2	2								
Swendseid et al. (2019) [26]	RCSS	18/24	2	2	0	2	0	2	2	0	2	2	2	2								
Wang et al. (2016) [11]	RCCS	16/24	2	2	0	2	0	2	2	0	2	0	2	2								
Zhang et al. (2015) [24]	RCCS	16/24	2	2	0	2	0	2	2	0	2	0	2	2								

RCS—retrospective case study; PCS—prospective case study; RCCS—retrospective case-controlled study. Case studies were scored according to the Institute of Health Economics (IHE) quality appraisal checklist for assessing case-series studies, as follows: 1 (yes) or 0 (partial/unclear/no). Case controlled studies were scored according to the Methodological Index for Non-Randomized Studies (MINORS) as follows: 0 (not reported), 1 (reported but inadequate), or 2 (reported and adequate).

References

Guo B, Moga C, Harstall C, Schopflocher D: A principal component analysis is conducted for a case series quality appraisal checklist. J Clin Epidemiol 69:199-207 e192, 2016.Slim K, Nini E, Forestier D, Kwiatkowski F, Panis Y,

Chipponi J: Methodological index for non-randomized studies (minors): development and validation of a new instrument. ANZ J Surg 73:712-716, 2003.
